# Editorial: Genomic strategies for efficient microbial cell factories

**DOI:** 10.3389/fbioe.2022.962828

**Published:** 2022-08-05

**Authors:** Eugene Fletcher, Yun Chen, Luis Caspeta, Amir Feizi

**Affiliations:** ^1^ Escarpment Laboratories, Guelph, ON, Canada; ^2^ Department of Biology and Biological Engineering, Chalmers University of Technology, Göteborg, Sweden; ^3^ Universidad Nacional Autonoma de Mexico, Cuernavaca, Mexico; ^4^ OMass Therapeutics, Oxford, United Kingdom

**Keywords:** yeast, design-build-test-learn (DBTL) cycle, diatoms, synthetic biology, xylanases, direct RNA sequencing, friedelin, biomanufacturing

Following a renewed commitment to build a sustainable bioeconomy, there has been a heightened shift in interest from the production of petroleum based chemicals to biomanufacturing. To make biomanufacturing competitive with chemical synthesis, it is important to develop microbial cell factories that can efficiently utilize cheap and readily available feedstocks. These feedstocks are then metabolized to yield value-added products in high titres that can be easily scaled up to commercial quantities. Advances in synthetic biology have paved the way for efficient microbial design and improvement to reduce the cost and time of feedstock bioprocessing. These cell factories can further be optimized by fine-tuning relevant metabolic pathways utilizing the iterative and systematic Design-Build-Test-Learn (DBTL) cycle (Carbonell et al., 2018). Currently, automation and machine learning technologies are being integrated into the DBTL cycle to increase the throughput, efficiency and turnaround time of developing efficient microbial strains (Carbonell et al., 2018). Also recently, the advent of sequencing and CRISPR/Cas technology enabled the development of a molecular toolbox to edit the genome of microbial hosts as a core principle of strain engineering, and fundamental to the DBTL cycle. In parallel, progress made in developing omics technologies has resulted in lots of data required to build robust genome-scale metabolic models which can be used to predict and optimize metabolic fluxes in microbial cell factories during biomanufacturing. Genome editing technologies have been well-established for a handful of microbial strains including bacteria and yeast (Yang & Blenner 2020; Arroyo-Olarte et al., 2021; Krogerus et al., 2021). Future work will apply these tools to non-conventional microorganisms that are more capable of producing specific value-added compounds.

**FIGURE 1:**
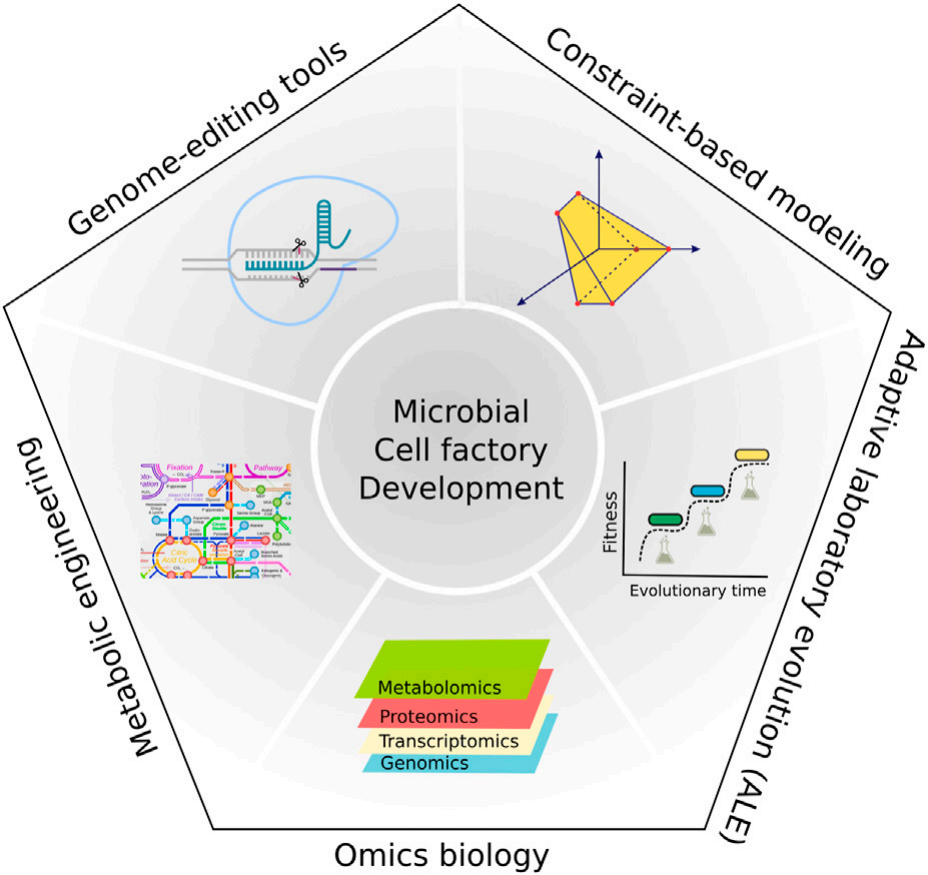
The schematic summarizes different genomic strategies that are highlighted in this research topic and are being used to develop microbial cell factories. An integration of these tools will be required to further improve microbial hosts used for biomanufacturing.

In this research topic, we highlight a broad range of reviews and original research covering several applications of genomic strategies (Figure 1) being applied to improve complex sugar consumption, and the production of non-native compounds in eukaryotic cell factories.

Transcriptomic data is essential in understanding global gene expression of microbial hosts under different condition (Caspeta et al., 2014; Fletcher et al., 2017). This data can be fed into genome-scale metabolic models and the DBTL cycle to make microbial cell factories more efficient. Therefore, it is imperative to make informed choices when selecting a suitable long-read sequencing method to study gene functions of cells under specific conditions. In this light, we highlight work published in this research topic by Wongsurawat et al. where the authors provide a comparative study of transcriptomic analyses using direct RNA sequencing (dRNA-seq) or direct cDNA sequencing (dcDNA) reads. While dRNA-seq is cheaper and simpler to perform, dcDNA-seq provides a higher accuracy as reads are longer.

As mentioned, development of genome editing tools continues to play a key role in genome-wide engineering of microbial hosts for the production of value added compounds. Gao et al. demonstrate the production of friedelin (an important plant triterpenoid compound with remarkable pharmacological activities) in *Saccharomyces cerevisiae* by integrating the endogenous pathway genes of friedelin into *S. cerevisiae*. To increase the friedelin yield, competing pathways were knocked out by applying the CRISPR/Cas9 system. Using this genomic strategy, Gao et al. were able to significantly increase the yield of friedelin produced which is the highest reported titre in yeast to date. It will be interesting to see future work on the production of such triterpenoid compounds from cheap feedstocks such as lignocellulose.

Although lignocellulose is cheap and readily available, industrially relevant microbial hosts such as *S. cerevisiae* are unable to utilize most of the sugars in hydrolyzed lignocellulose. Xylose which is abundant in lignocellulose cannot be metabolized as a sole carbon source by *S. cerevisiae* unless a non-native xylose isomerase and xylose reductase pathway is engineered in this yeast (Van Maris et al., 2007). To address this challenge, an alternative approach is required to improve the utilization of carbohydrate polymers such as xylans in *S. cerevisiae*. Procopio et al. review the current state of xylan (xylose polymer) metabolism in yeast where the focus is on engineering *S. cerevisiae* to assimilate these polymers to produce bioethanol. Both extracellular utilization through cell surface engineering and intracellular transport followed by polymer degradation have been exploited. In some cases, engineering *S. cerevisiae* to express non-native xylanases from bacteria and other fungi has been reported to result in a non-specific metabolic burden. Hence, complementing rational engineering with adaptive laboratory evolution presents a promising genomic strategy for efficient yeast engineering to convert complex lignocellulosic sugars into bioethanol.

Lastly, expanding the repertoire of high-value compounds produced by microbial cell factories will require exploiting non-conventional microbial hosts in order to tap into the unique biochemistries of their metabolic pathways. In that regard, microalgae such as diatoms offer excellent opportunities that have not been fully explored. Having an innate ability to produce large amounts of lipids (Yi et al., 2017), diatoms serve as perfect hosts for the production of biofuels and bioactive compounds such as carotenoids and fatty acids. The production of non-native compounds with diatoms is, however, limited by lack of genetic tools required to engineer its genome. Chen et al. highlight the potential application of genome engineering tools such as CRISPR in diatoms. Furthermore, the first sequenced genome of a diatom is discussed which has made it possible to build genome-scale metabolic models for diatoms. In order to fast track the development of diatoms as suitable microbial cell factories, future work should go into developing high quality genome annotations essential for building more comprehensive metabolic models in diatoms. In well-studied organisms like *E. coli* and *S. cerevisiae*, genome-scale metabolic models have played a key role in guiding metabolic engineering strategies (Gu et al., 2019). Thus, metabolic engineering of diatom cell factories will benefit from such metabolic models.

We conclude by emphasizing that the articles presented in this research topic provide a glimpse of the role of genome-wide tools in making microbial cell factories more efficient at producing high titres of desired biochemicals. We anticipate that these studies will inspire the development of new genomic tools as well as the improvement of existing tools for microbial strains design and engineering. Ultimately, improved microbial performance will enhance biomanufacturing and fast track the efforts towards creating a sustainable and environmentally-friendly society.
